# Enhancing Pixel Charging Efficiency by Optimizing Thin-Film Transistor Dimensions in Gate Driver Circuits for Active-Matrix Liquid Crystal Displays

**DOI:** 10.3390/mi15020263

**Published:** 2024-02-10

**Authors:** Xiaoxin Ma, Xin Zou, Ruoyang Yan, Fion Sze Yan Yeung, Wanlong Zhang, Xiaocong Yuan

**Affiliations:** 1 Nanophotonics Research Center, Institute of Microscale Optoelectronics, Shenzhen University, Shenzhen 518060, China; 2 College of Electronics and Information Engineering, Shenzhen University, Shenzhen 518060, China; 3 State Key Laboratory on Advanced Displays and Optoelectronics Technologies, Department of Electronic & Computer Engineering, Hong Kong University of Science and Technology, Hong Kong SAR, China

**Keywords:** thin-film transistors (TFTs), active-matrix liquid crystal displays (AMLCD), pixel charging ratio, gate driver on array (GOA) circuit

## Abstract

Flat panel displays are electronic displays that are thin and lightweight, making them ideal for use in a wide range of applications, from televisions and computer monitors to mobile devices and digital signage. The Thin-Film Transistor (TFT) layer is responsible for controlling the amount of light that passes through each pixel and is located behind the liquid crystal layer, enabling precise image control and high-quality display. As one of the important parameters to evaluate the display performance, the faster response time provides more frames in a second, which benefits many high-end applications, such as applications for playing games and watching movies. To further improve the response time, the single-pixel charging efficiency is investigated in this paper by optimizing the TFT dimensions in gate driver circuits in active-matrix liquid crystal displays. The accurate circuit simulation model is developed to minimize the signal’s fall time ($T_{f}$) by optimizing the TFT width-to-length ratio. Our results show that using a driving TFT width of 6790 μm and a reset TFT width of 640 μm resulted in a minimum $T_{f}$ of 2.6572 μs, corresponding to a maximum pixel charging ratio of 90.61275%. These findings demonstrate the effectiveness of our optimization strategy in enhancing pixel charging efficiency and improving display performance.

## 1. Introduction

Active-matrix (AM) [1] displays, including AM liquid crystal displays (AMLCDs) [2] and AM organic light-emitting diode displays (AMOLEDs) [3], have become the leading products in the flat panel display market [4]. Thin-Film Transistors (TFTs) are essential to these displays and are continuously improved for enhanced performance and reliability. However, as LCD resolution improves, a significant challenge arises: the diminished pixel charging ratio. The reduction in charging time is a critical bottleneck that affects the overall response time and display quality, especially in high-frequency refresh AMLCDs. The need to maintain high image quality at higher resolutions and refresh rates has driven extensive research and development in TFT technology. Innovations have focused on enhancing TFT materials, refining circuit layouts, and boosting the operational efficiency of the pixel-driving mechanisms. In modern display applications, clarity, color accuracy, and refresh rate are critical for delivering an optimal viewing experience that matches the demanding standards of modern visual applications.

To address the challenge of improving pixel charging efficiency, the industry has evolved in various areas, including the enhancement of digital to analog converters (DAC), the development of new pixel driving circuits, and advancements in TFT device design [5,6,7,8,9]. These innovations have been crucial in addressing the limitations of current display technologies. Notably, Seo et al. improved the DAC structure by increasing the timing controller’s input data from 8 to 10 bits, thereby augmenting the charging ratio in high grayscale ranges through pixel overdrive methods [10]. Chen et al. proposed to simplify the amorphous silicon (a-Si) gate drive circuit by reducing the clock duty cycle, which saves layout space and boosts the charging ratio [11]. Furthermore, Chiang et al. suggested enhancing the drive current by increasing the gate input signal voltage to improve gate drive efficiency [12]. Recent developments have shown promising results in using low-temperature polycrystalline silicon (LTPS) TFTs for their superior electron mobility, aiming to improve pixel charging in high-resolution displays [13]. Advancements in amorphous indium–gallium zinc oxide (a-IGZO) TFTs have been pivotal in achieving stable pixel circuits for AMOLEDs, particularly in mobile devices [14]. These advancements collectively represent a significant leap in TFT technology, aiming to overcome the challenges posed by higher resolution and refresh rates in modern displays.

In this paper, we introduce a novel approach to enhance pixel charging efficiency in AMLCDs by modifying the device structure within the gate drive circuit, maintaining the integrity of the pixel drive circuit’s original design. We detail the operational principles of the 11T1C (11 transistor 1 capacitor) gate driver on the array (GOA) circuit and analyze the gate drive circuit’s output dynamics, particularly focusing on how the fall time ($T_{f}$) affects pixel drive circuit performance. Using hydrogenated a-Si TFTs, our comprehensive simulations revealed a notable enhancement in pixel charging efficiency, primarily driven by the reduction of the gate drive circuit’s output signal $T_{f}$. This discovery underscores the critical impact of $T_{f}$ on display quality and offers a novel lens through which to view circuit optimization. Additionally, we determined an optimal device layout, balancing the demands of spatial efficiency and performance optimization. By maintaining the GOA circuit’s unit layout area at 1200 μm×199.8 μm, we not only adhered to standard design norms but also achieved a remarkable charging efficiency of up to 90.61275%.

## 2. Materials and Methods

In field-effect transistors, the drain-source current $I_{ds}$ is critically influenced by two voltages: the voltage between source and drain, $V_{DS}$, and the voltage between gate and source, $V_{GS}$. The relationship is essential for grasping transistor operations and is described in the following equation [15]:

(1) $$I_{ds}=\frac{W}{L}\mu C_{g}(V_{GS}-V_{T}-\frac{V_{DS}}{2})V_{DS}.$$

In this equation, *W* and *L* denote the channel width and length, respectively, demonstrating how the channel’s physical dimensions impact current flow of the transistor. $C_{g}$ is the gate capacitance and is pivotal in controlling charge accumulation at the gate. $V_{T}$ denotes the threshold voltage, which is the minimum gate voltage to establish a conductive path between the source and drain. The mobility of the majority carrier within the TFT channel, represented by μ, dictates the ease with which charge carriers traverse the channel. Assuming $\beta =\frac{W}{L}\mu C_{g}$, the expression for $I_{ds}$ can be further simplified to [16]:

(2) $$I_{ds}=\beta (V_{GS}-V_{T}-\frac{V_{DS}}{2})V_{DS}.$$

This streamlined equation offers a simpler method for calculating the drain-source current despite being less detailed. It accurately captures the impact of crucial variables, including gate-to-source voltage, drain-to-source voltage, channel dimensions, carrier mobility, and gate capacitance.

### 2.1. Pixel Circuit

**Figure 1:**
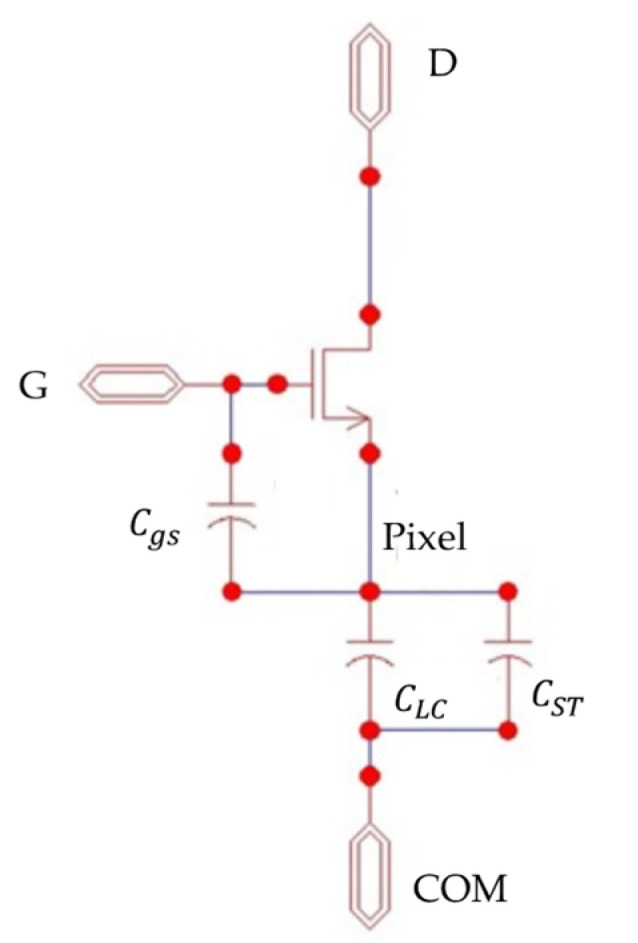
Schematic of pixel driving circuit.

Since the anisotropic liquid crystal molecules in LCDs are torqued in a specific direction by the alignment layer, with an external electric field applied, the molecules’ orientation is moved either parallel or cross to the electric field concerning the dielectric polarity of the molecules. The reorientation of the liquid crystal molecules allows light to pass through or be blocked under cross polarizers, creating the full-color display information with individual sub-pixels with red, green, or blue color filters. Figure 1 illustrates the novel pixel structure, consisting of a TFT and two capacitors. The two main phases of the AMLCD pixel circuit are charging and holding [17]. During the high-level state of the gate signal G, the TFT activates and allows the data signal D to enter the liquid crystal capacitor $C_{LC}$, thereby charging the pixel voltage. Conversely, when G is positioned at a low-level state, the TFT deactivates, maintaining the pixel’s voltage through the storage capacitor $C_{ST}\;$. This modification in voltage leads to a change in the liquid crystal molecules’ orientation, modulating light transmittance and creating the display information. Thus, during the row-on period, the pixel voltage $V_{pixel}(t)$ can be given by [18]:

(3) $$V_{pixel}(t)=(V_{DH}-V_{DL})[1-exp(\frac{-t}{R_{on}C_{pixel}})],$$

where $V_{GH}$ and $V_{GL}$ denote row scanning signal’s high and low levels, respectively, while $V_{DH}$ and $V_{DL}$ represent the data signal’s high and low levels, respectively. $R_{on}$ is the on-state resistance, and $C_{pixel}$ is the total capacitance, combining storage and liquid crystal capacitances. The charging ratio is defined as follows:

(4) $$Charging\;Ratio=\frac{V_{pixel}}{\left(V_{DH}-V_{DL}\right)}*100\%.$$

From Equations (3) and (4), we can find that during the row-on period, the charging ratio is directly proportional to the charging duration t.

To prevent erroneous signals in the pixel unit, it is necessary to apply delay compensation to the signal timing [11]. The preferred method involves prematurely closing the scan line, ensuring that the gate signal G falls before the corresponding signal D. If G falls earlier than D by a margin exceeding the $T_{f}$ time, this guarantees that the TFT is off when D begins to fall, thus ensuring accurate circuit output. While this approach guarantees that the TFT shuts off before the signal line data switch to the next scan line, it consequently reduces the available charging time for the pixel during the charging interval, leading to a decreased charging rate. Therefore, decreasing the TF value can extend the circuit’s actual charging time, enhancing the charging rate. This relationship demonstrates the inverse proportionality between the charging rate and $T_{f}$.

### 2.2. Gate Driver Circuit

**Figure 2:**
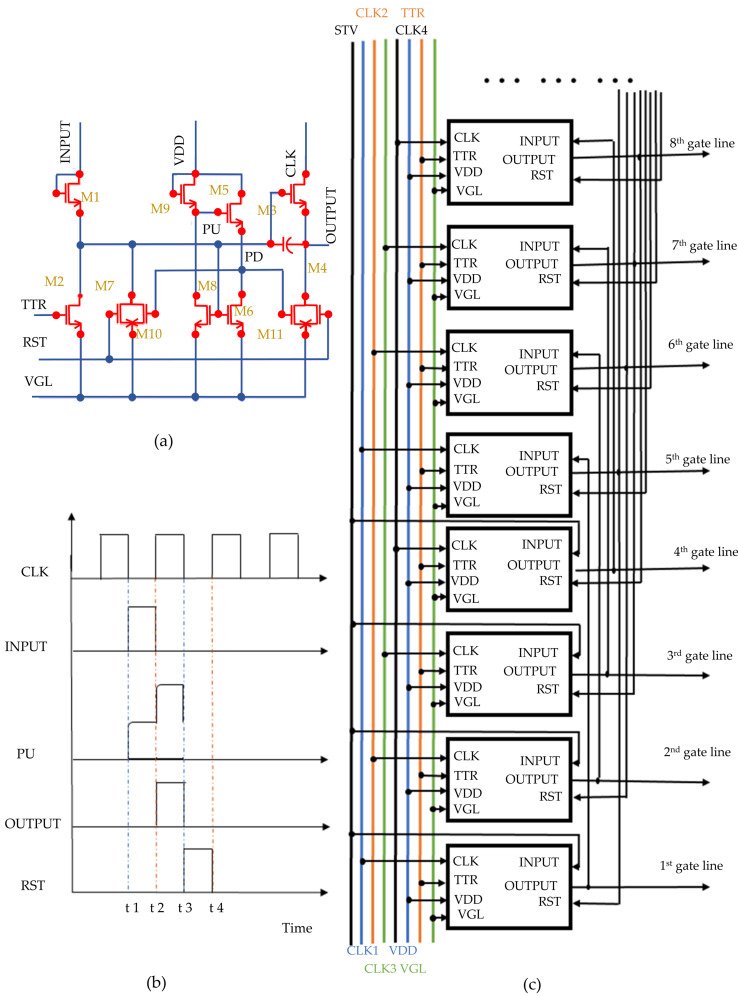
A-Si TFT gate driver with (**a**) single-stage circuit, (**b**) timing diagram, and (**c**) block diagram.

Figure 2 presents the schematic of the gate driver used in this study, consisting of three distinct sections [19]: (a) the single-stage circuit schematic, (b) the timing diagram, and (c) the block diagram. The single-stage circuit (a) features a capacitor C1 alongside 11 TFTs (M1–M11) [20], where M1 is the input TFT, M3 is the driving TFT, and M2, M4–M10 are the reset TFTs. This circuit operates through four phases: Pre-Charge Period, Pull-Up Period, Pull-Down Period, and Low-Level Holding Period [21,22]. Within this circuit, the PU node acts as the pull-up point to control the OUTPUT signal, while the PD node serves as the pull-down point. This configuration enables the progressive scan-driving function of the LCD panel. The scanning drive circuit operates as a shift register, generating a shift pulse signal as the OUTPUT signal in response to external control signals. This OUTPUT signal simultaneously activates the TFTs in the current row and also functions as the initiation signal for the next row and the termination signal for the previous row, presenting the driving principle of the novel liquid crystal panels.

From t1 to t2, with the INPUT signal at a high level and both RST and CLK at a low level, M1 activates. This transition raises $V_{PU}\;$at node PU to $V_{PU1}$, representing the gate level of M3. With CLK low, M3 remains off. Meanwhile, M6 and M8 activate, lowering the PD node’s voltage, while M10 and M11 are off [11,23].

From t2 and t3, the CLK signal attains a high level, INPUT shifts from high to low, and RST maintains a low level. The capacitive coupling through the parasitic capacitance between the gate and source of M3 leads to an increase in the voltage at the PU node to $V_{PU2}$. This elevation enhances M3’s conduction capability, resulting in an increase in the voltage of the OUTPUT signal $V_{o}$ due to M3’s charging. It is important to recognize that the current GOA unit’s OUTPUT signal serves as the input for the subsequent stage. During this transmission, the next stage undergoes pre-charging.

From t3 to t4, the RST signal shifts from low to high level, while CLK shifts from high to low. M7 and M4 are then activated, pulling down the voltages at PU and OUTPUT points to $V_{GL}$. It is crucial to note that the PU point’s voltage does not diminish instantaneously, assisting in M3′s activation to expedite the OUTPUT signal’s discharge. The primary discharge occurs across the terminals of capacitor C, between the OUTPUT signal and the $V_{GL}$ potential. 

After t4, to maintain the OUTPUT’s low state prior to the next ascending phase, M6 and M8 deactivate. M9 and M5 conduct, keeping PD high and M10 and M11 off, stabilizing the capacitor’s voltage at $V_{GL}$.

The $T_{f}$ is predominantly influenced by the discharge path’s resistance R and the output node’s capacitance C. Regarding the discharge phase, both M3 and M4 significantly affect the OUTPUT node’s discharge [24], thus being integral to the OUTPUT signal’s $T_{f}$. We posit that the high level of the CLK signal is $V_{H}$, the low level is $V_{L}$, the INPUT signal’s voltage is $V_{in}$, and the RST signal’s voltage is $V_{R}$. 

For transistor M3, the equivalent resistance *R_M_*_3_ can be approximated as follows [25]:

(5) $$R_{M3}\approx \frac{1}{\beta_{3}(V_{GS3}-V_{T3})},$$

where $V_{GS3}$ denotes the voltage difference between the gate and source, which is pivotal for controlling the transistor’s conductance. $V_{T3}$ is M3’s threshold voltage, defined as the minimum voltage required to activate the transistor. Furthermore, $C_{PU}$ signifies the overlap capacitance at the PU point and $R_{M7}$ denotes M7′s equivalent resistance, which plays a crucial role in determining the discharge path resistance during the high-to-low transition of the CLK signal. During the CLK signal’s high-to-low transition, the voltage at node PU changes from $V_{PU2}$ to $V_{PU3}$, been calculated by

(6) $$V_{PU3}\approx V_{PU2}-(V_{H}-V_{L})\frac{C_{GD}}{C_{GD}+C_{GS}+C_{S}}.$$

From Equations (5) and (6), we derive

(7) $$R_{M3}\approx \frac{1}{\beta_{3}[(V_{PU3}-V_{L})(1-\frac{t-t3}{C_{Q}R_{M7}})-V_{T3}]}.$$

The average value of $R_{M3}$ is

(8) $$\bar{R_{M3}}\approx \frac{1}{\beta_{3}[(V_{PU3}-V_{L})(1-\frac{t_{a}}{C_{Q}R_{T7}})-V_{T3}]},$$

where $t_{a}$ is a fitted parameter. 

For M4, if $V_{GS4}-V_{T4}$ equals γ multiplied by $\left(V_{H}-V_{L}-V_{T4}\right)$, the average value of $R_{M4}$ is

(9) $$\bar{R_{M4}}\approx \frac{1}{\gamma \beta_{4}(V_{H}-V_{L}-V_{T4})}.$$

As M3 and M4 jointly provide a discharge path for OUTPUT, their parallel resistance is determined by

(10) $$\frac{1}{R_{total}}=\frac{1}{\bar{R_{M3}}}+\frac{1}{\bar{R_{M4}}}.$$

Considering $R_{L}$ and $C_{L}$ as the connected resistor and capacitor to the OUTPUT node, respectively, the $T_{f}$ simplifies to

(11) $$T_{f}=2.2C_{L}(R_{L}+R_{total})=2.2C_{L}(R_{L}+\frac{1}{\frac{1}{\bar{R_{M3}}}+\frac{1}{\bar{R_{M4}}}}).$$

Equations (8), (9) and (11) suggest that $T_{f}$ is inversely proportional to M3 and M4′s channel widths, indicating that increasing their widths can effectively reduce $T_{f}$.

## 3. Results and Discussions

**Figure 3:**
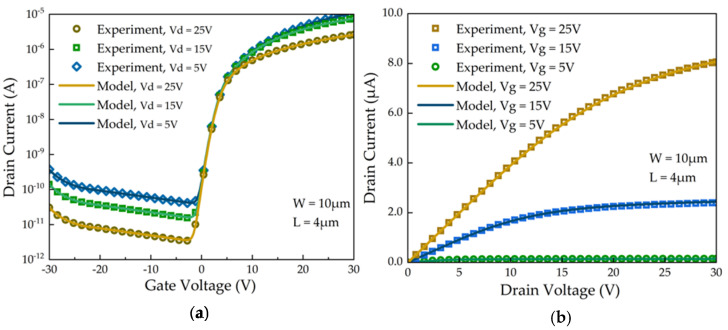
(**a**) The output characteristics and (**b**) the transfer characteristics of the a-Si TFTs.

To develop precise circuit models, we performed detailed feature extraction on a-Si TFT devices used in pixel-driving circuits. These extracted features are critical for subsequent simulation and verification processes. Figure 3a,b display the parameter extraction results for these TFT device models. Specifically, Figure 3a shows the transfer characteristic curve of a-Si TFT, and Figure 3b presents its current characteristic curve. The linear root mean square error (LinRMS) values in the linear and saturation regions are 0.92% and 1.59%, respectively. Moreover, Figure 3b demonstrates the electrical characteristics of a-Si TFTs in off, transition, and on states, with LinRMS values for these states being 2.27%, 2.81%, and 0.82%, respectively. The pixel-driving circuit investigated in this study incorporates a TFT device with a channel width of 10 µm and a channel length of 4 µm.

**Figure 4:**
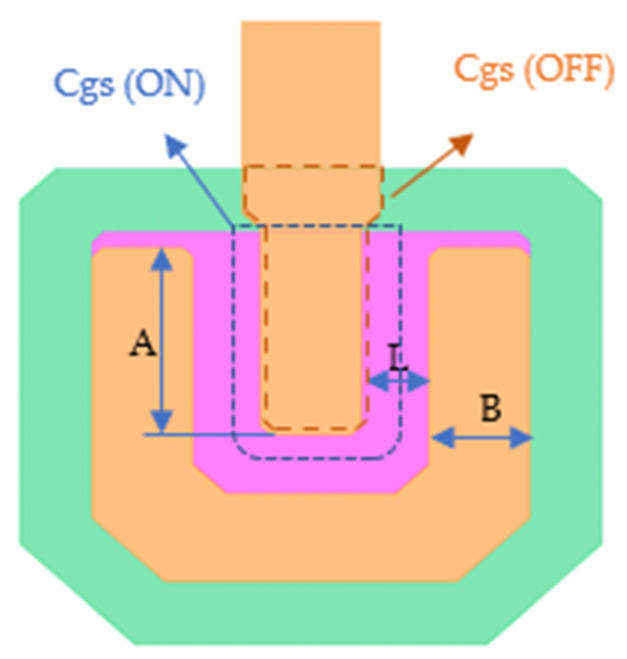
Structure of U-shaped TFT.

Figure 4 presents a U-shaped TFT design. This design efficiently increases the channel width W while minimally enlarging the total TFT area. Due to its resemblance to the letter ‘U’, the channel is termed U-shaped. This design strategy balances the control of both the area and the $C_{gs}$ of the TFT. In the on state, the $C_{gs}$ area, outlined by a blue dotted line and determined by the gate insulating layer’s thickness $\;t_{ox}$ between the gate metal and the active layer is visible. In contrast, during the off-state, the $C_{gs}$ area, marked by a red dotted line, predominantly overlaps the region between the drain and gate metals. The thickness of this area is represented by the combined thickness of both metal layers, expressed as $\;t_{ox}+t_{si}$.

The channel width W of the U-shaped TFT is determined using the equation:

(12) W=2A+B,

where *A* and *B* represent direct layout measurement values, as shown in Figure 4. The design parameters of the pixel drive circuit, as shown in Table 1, are derived from the extracted values of capacitance and resistance.

In this study, we aim to engineer a display with a resolution of 1680 × 320 pixels, focusing on optimizing the pixel charging ratio. We rigorously investigate the cascade effect in the gate drive circuit and assess the impact of scan electrode signal delay on display performance. This methodology underscores our dedication to improving display quality by meticulously balancing technical precision and efficiency through comprehensive circuit analysis and optimization strategies. For this analysis, a total of nine waveforms are selected on pixels at Column 1, Column 840, and Column 1680 of Rows 2, 180, and 360. In this selection, $T_{f}\;$denotes the maximum delay time among the nine waveforms, and the “charge ratio” indicates the lowest charge ratio among the chosen pixels. The layout area for the GOA is 1200 μm×199.8 μm. The channel width *L* of all TFTs is standardized at 3.5 μm. Utilizing Equation (12), the actual channel width is calculated as

(13) W=(n+1)×(A−0.7)+n×(B−0.7),

where *n* is the number of U-shaped structures.

For M3 and M4’s channel width calculations, with *n* varying from 7 to 39 (an integer), the formulas are

(14) $$W_{M3}=(n+1)(175.83-0.7)+n(4.2-0.7),$$

(15) $$W_{M4}=(41-n)(157.45-0.7)+(40-n)(4.2-0.7).$$

For M1, the calculation yields

(16) $$W_{M1}=3\times \left(93.65-0.7\right)+2\times \left(4.2-0.7\right)\approx 284\;\mu m.$$

For M2 and other transistors (M5 to M11), the calculation yields the following:

(17) $$W_{M2,M5,M6,M7,M8,M9,M10,M11}=2\times \left(8.95-0.7\right)+\left(4.2-0.7\right)=20\;\mu m.$$

**Figure 5:**
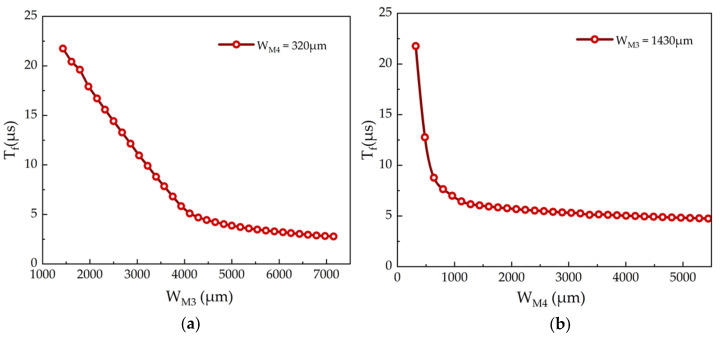
The relationship between $T_{f}$ and channel widths of (**a**) M3 and (**b**) M4, respectively.

These calculations facilitate the determination of dimensions corresponding to various channel widths, enabling further layout design and analysis. The OUTPUT signal primarily originates from transistor M3, making it the most directly associated TFT with the OUTPUT signal. Simultaneously, the conduction of M4 significantly reduces the OUTPUT signal, affecting the fall time $T_{f}$. Other TFTs in the circuit mainly function as switches or for noise reduction. Figure 5a,b illustrate the relationship between the $T_{f}$ and the channel widths of M3 and M4, respectively. The simulation results indicate an inverse relationship between $T_{f}$ and $W_{M3}$: $T_{f}$ decreases as $W_{M3}$ increases from 1430 µm to 7150 µm. Similarly, with $W_{M3}$ fixed at 1430 µm, an increase in $W_{M4}$ from 320 µm to approximately 1000 µm results in a reduction of $T_{f}$. This finding is consistent with Equation (12). 

Increasing the channel widths of M3 and M4 raises their parasitic capacitance, resulting in greater capacitive reactance during signal transmission. However, a larger channel width substantially enhances their conduction ability while active. Thus, for M3 and M4, which initially have narrow channels, widening the channel width improves conduction during operation and effectively shortens the fall time. Balancing $W_{M3}$ and $W_{M4}$ is crucial for optimizing efficiency and performance to minimize $T_{f}$.

**Figure 6:**
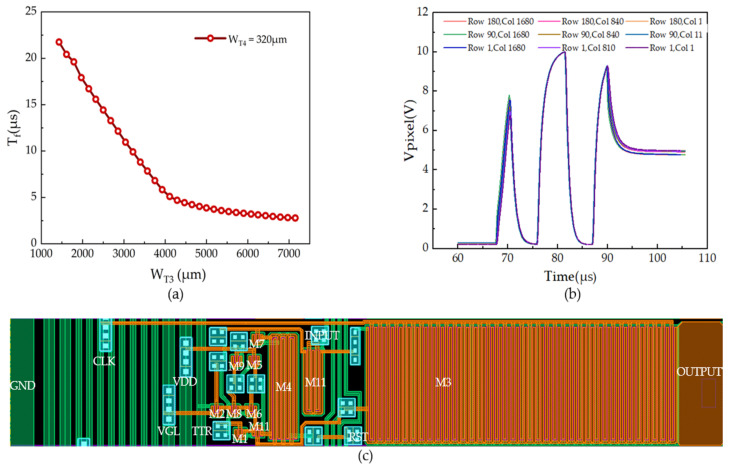
(**a**) The charging ratio and signal’s fall time $T_{f}$ curves versus the channel width of M3, with a fixed GOA layout of 1200 µm × 199.8 µm; (**b**) normalized pixel voltage waveforms at the selected nine locations, configurations $W_{M3}$ = 6790 µm, $W_{M4}$ = 640 µm; (**c**) layout structure of a single GOA circuit.

In our study, the optimal widths for M3 and M4 are established through a series of simulation experiments under fixed layout design parameters. These simulations demonstrate that a width of 6790 µm for M3 and 640 µm for M4 achieve the shortest OUTPUT signal $T_{f}$ and the highest pixel charging ratio. Specifically, the $T_{f}$ of the OUTPUT signal is recorded at 2.6572 µs, while the pixel charging ratio reaches 90.61275%. The configuration of M3 and M4 transistors influences the $T_{f}$ value and charging ratio, as presented in Figure 6a. The experiment is conducted within a constant layout area, and the results show that as the channel width of M3 increases, the channel width of M4 correspondingly decreases. However, it is not simply a case of “the larger, the better” for the M3 channel width. The optimal charging rate occurs when M3’s width is 6790 µm and M4’s width is 640 µm. Further increasing M3’s channel width beyond this point leads to a reduction in the charging ratio. The essence of achieving the highest charging ratio lies not solely in maximizing dimensions but in striking a delicate balance that ensures the most efficient charging process. 

Figure 6b illustrates the time-dependent variation of pixel voltage waveforms at the selected nine positions with the configurations $W_{M3}$ of 6790 µm and $W_{M4}$ of 640 µm. An examination of the figure shows that during the charging process, the waveform experiences three distinct peaks [26]. The initial two peaks correspond to the circuit’s “warm-up” charging phase, designed to eliminate residual charge in the parasitic capacitance from the previous cycle, thus avoiding interference from past charges. The first pre-charge phase clears any remaining charge, while the second sets a clear threshold or reference for subsequent operations or display cycles. The importance of the “warm-up” charging phase lies in its role in removing residual charge and avoiding interference from prior charges, ensuring stable and precise operation. The presence of parasitic capacitances in the OUTPUT signal and the GOA unit causes attenuation of the CLK signal during transmission, resulting in insufficiently charged voltage for subsequent OUTPUT signals. To address this issue, multiple CLK designs are commonly implemented. The intricate design and strategic implementation of these multiple CLK lines, coupled with careful consideration of the effects of parasitic capacitance, are critical in improving display performance. This holistic approach to circuit design guarantees efficient charging, stability, and consistency of the display output. Moreover, Figure 6b also presents the consistency of the normalized pixel voltage waveforms at the selected nine locations, proving the robustness of this practical design. 

In the primary charging phase, the voltage rapidly increases, with readings at all observed points exceeding 9.2391 V and a charging ratio over 90.61275% under the simulation parameters listed in Table 2. This design also ensures that future signal charging or modulations start from this established value, thereby improving the consistency and stability of signal processing. Figure 6c displays the layout structure of a single GOA circuit, highlighting the organization of TFT within the circuit. To achieve a higher charging ratio within a constrained layout area, the strategic placement of each TFT and via is crucial to optimize space utilization. This design prioritizes the ample allocation of space for M3 and M4, while other TFTs, vias, and traces are meticulously organized to comply with layout design principles and conserve space. Two approaches are adopted to minimize the area used for wiring between TFTs: firstly, grouping TFTs with dual-end connections, such as pairing M5 with M9, M10 with M11, and M6 with M8, along with M7 and M4; secondly, designing non-resistor-value-optimization-related traces to be as narrow as possible. This systematic approach to layout maximizes space efficiency, thereby improving the charging ratio in the GOA layout. Increasing the linewidth of the CLK signal line positively influences the OUTPUT signal of the GOA unit, primarily by reducing its $T_{f}$. Therefore, in situations with limited layout space preventing the further expansion of the TFT channel width, reallocating space to enlarge the CLK signal line’s linewidth is an effective strategy. Such modifications ensure the judicious use of a confined space, contributing significantly to the circuit design’s overall optimization. Additionally, the layout ensures the even distribution of VDD, GND, CLK, TTR, and VGL signals throughout the display panel, underscoring the thoroughness of the design to achieve optimal performance.

## 4. Conclusions

In this study, we conduct a comprehensive analysis of the substantial impact that driving and resetting TFTs have on the $T_{f}$ of GOA output signals in AMLCDs. The accuracy of extracting model parameters and identifying parasitic components of TFTs is crucial for precise circuit simulations. Our extensive simulations and experiments demonstrate that modifying the width-to-length ratio of TFTs within the constraints of the GOA cell layout can markedly reduce $T_{f}$. The implementation of an optimized driving TFT width of 6790 µm and a reset TFT width of 640 µm achieve a minimum$\;T_{f}$ of 2.6572 µs and a maximum charging ratio of 90.61275% in our simulations, confirming the significance of TFT dimension optimization for enhancing the pixel charging ratio, indicating the improvement of the TFT driving circuit for high-frame-rate display devices.

## Figures and Tables

**Table 1:** Design parameters of the pixel circuit.

Parameters	Value (fF)	Parameters	Value (µm)
$C_{gs}$	31.377	$W_{pixel}$	10
$C_{LC}$	18.717	$L_{pixel}$	4
$C_{ST}$	168.456		

**Table 2:** Design parameters of the GOA circuit.

Parameters	Value	Parameters (Unit)	Value
$W_{M1}$	284 µm	INPUT	−8–22 V
$W_{M2}$	20 µm	CLK	−8–22 V
$W_{M3}$	6850 µm	CLK	−8–22 V
$W_{M4}$	640 µm	VGL	−8 V
$W_{M5-M11}$	20 µm	VDD	22 V
$L_{M5-M11}$	3.5 µm	TTR	−8–22 V
$C_{1}$	4 pF		

## Data Availability

Data are contained within the article.
